# A Cross-Sectional Evaluation of Cardiovascular Risk Assessment in Type 2 Diabetes Mellitus Patients Using the Framingham Risk Score

**DOI:** 10.7759/cureus.58026

**Published:** 2024-04-11

**Authors:** Mitali B Rathod, Shashum Moukthika, Allwyn John Karikunnel, Kottala Harika, Prathibha Talla, Madhurika Jalakam

**Affiliations:** 1 Department of General Medicine, NAMO Medical Education and Research Institute, Silvassa, IND; 2 Department of General Medicine, Makerere University, Kampala, UGA; 3 Department of Accident and Emergency, Milton Keynes University Hospital, Milton Keynes, GBR; 4 Department of General Medicine, ONE Hospitals, Karimnagar, IND; 5 Department of General Medicine, KIMS (Krishna Institute of Medical Sciences) Hospitals, Ongole, IND; 6 Department of General Medicine, MNR Medical College and Hospitals, Hyderabad, IND

**Keywords:** smoking, hypertension, males, framingham cardiovascular risk score, cardiovascular disease, type 2 diabetes mellitus

## Abstract

Background: The global burden of type 2 diabetes mellitus (T2DM) is continuously increasing, particularly in India. The risk of cardiovascular disease (CVD) is higher in T2DM individuals when compared to non-diabetics, which imposes significant morbidity and mortality. The main aim of the present study was to assess the risk factors for CVD in T2DM patients. The secondary aim was to assess the association between cardiovascular risk profile and 10-year cardiovascular risk using the Framingham risk score.

Methods: This was a cross-sectional study conducted on 110 T2DM patients, and the anthropometric and biochemical parameters were analyzed. The Framingham cardiovascular risk prediction model was used to calculate the 10-year risk for CVD. The CVD risk factor was compared among the genders. Further, the association between the Framingham cardiovascular risk and the various categories of risk factors was also analyzed.

Results: Based on the Framingham cardiovascular risk score, 23 (20.9%) were at low risk, 39 (35.5%) were at moderate risk, and 48 (43.6%) were at low risk. A higher proportion of males had hypertension (55.2 vs. 17.3%; p=0.007), elevated cholesterol levels (48.3% vs. 23.1%; p=0.008), and smoking or tobacco use (31% vs. 7.7%; p=0.006) as compared to females. The significant risk factors for high 10-year CVD risk were hypertension (p=0.001), elevated total cholesterol (p=0.03), smoking or tobacco use (p=0.007), and glycemic control (p=0.04).

Conclusion: The Framingham cardiovascular risk score estimates reveal that male gender, hypertension, smoking, and uncontrolled diabetes are the important risk factors for CVD progression among diabetic patients. Therefore, it is imperative to generate awareness regarding the potential risks and then implement suitable interventions during the early phases at the primary healthcare level.

## Introduction

Diabetes mellitus (DM) is a chronic disease that results from an increased blood glucose level. It is due to deficient insulin production or a failure in insulin utility. Type 2 diabetes mellitus (T2DM) is the most common type of diabetes, accounting for 90% of the global population. According to the International Diabetes Federation (IDF)'s report for 2021, 540 million people worldwide have diabetes, which affects about 10.5% of adults (20-79 years old) [1]. India accounts for one in seven of all adults living with diabetes globally. The prevalence rate of T2DM in India for the year 2021 is reported to be 9.6% [1].

Atherosclerotic cardiovascular disease (ASCVD) is notably high in India as compared to Western countries. It is well known that the onset is 10 years earlier in Indians than in the Western population [2]. According to global burden estimates from 2019, it has been observed that the prevalence of age-standardized years of life lost due to CVD in South Asia is at least twice as high as in Western Europe and Australia [3]. The burden of ASCVD is higher in T2DM patients, with nearly 75% of the mortality due to this condition [4].

T2DM is associated with a significantly increased risk of vascular events, including coronary artery disease, ischemic stroke, and vascular mortality. This risk is independent of other risk factors and is mostly higher for women compared to males, particularly when diabetes is diagnosed at an early stage [5]. The decrease in life expectancy is 10 years, and CVD is the primary factor contributing to mortality among individuals with T2DM. T2DM patients experience a significant impact from CVD compared to those without diabetes. The mortality rates seen in individuals with diabetes were found to be 15.4% for those who had not experienced a prior myocardial infarction (MI) and 42% for those who had a history of MI. In contrast, patients without T2DM exhibited cardiovascular mortality rates of less than 2.1% and 15.9%, respectively [4]. In order to determine a patient's risk of CVD, it is necessary to have knowledge of the cardiovascular profile of patients with T2DM and the risk prediction model [6]. The risk of CVD in T2DM exhibits a gradual increase, and the intensity is influenced by a combination of several risk factors. The majority of these supplementary hazards of CVD in T2DM are linked to a higher occurrence of established risk factors, such as hypertension, dyslipidemia, and obesity [7]. In recent years, research has demonstrated that addressing identified risk factors for individuals with T2DM is crucial in mitigating the likelihood of CVD and also for adequate management. In this backdrop, the present study was conducted to evaluate the cardiovascular risk factor and its association with the Framingham risk score for the prediction of CVD in T2DM patients.

## Materials and methods

This was a cross-sectional study conducted on 110 T2DM patients using the random sampling method, aged between 35 and 75 years, who were the outpatients of the Department of General Medicine, NAMO Medical Education and Research Institute, Silvassa. The study was conducted for a period of one year, from November 2022 to December 2023, to assess the CVD risk in T2DM patients and its risk factors. The diabetes was diagnosed based on the American Diabetes Association criteria with fasting blood glucose (FBG) levels ≥126 mg/dL [8]. The study was approved by the Institutional Ethical Committee of NAMO Medical Education and Research Institute (approval number: IEC/2022/427/282). Before the data collection, the study procedure was explained to the patients and the caregivers, and informed consent was obtained from all the participants.

Inclusion criteria

Individuals diagnosed with T2DM who were aged between 35 and 75 years and had a duration of onset over one year were included in the study.

Exclusion criteria

Individuals with type 1 diabetes mellitus (T1DM), gestational diabetes, and pre-existing cases of coronary artery disease or stroke and those who declined to participate were excluded from the study.

Study procedure

Detailed medical history of the patients such as age, gender, educational level, income, smoking habits, hypertension, family history of DM and CVD, duration of DM, level of physical activity, and type of anti-glycemic medication used or employed (oral hypoglycemic agents, insulin therapy) were recorded.

Anthropometric measurements were conducted with the established methodologies outlined by Weiner and Lourie [9]. The standing height was measured with a set precision of 0.1 cm and fixed with a stadiometer. The measurement of body weight was done using a weighing scale with a precision of 0.5 kg. The waist circumference (WC) and hip circumference were measured using flexible steel tape, with measurements taken to the nearest 0.1 cm. The calculation of body mass index (BMI) involves dividing the total body weight by the square of the height (kg/m^2^). The waist-hip ratio (WHR) and waist-height ratio (WHtR) were also calculated. The patients were classified based on their BMI using the World Health Organization (WHO) classification. Central obesity was evaluated by employing the conventional criteria of WC, WHR, and WHtR.

The systolic blood pressure (SBP) and diastolic blood pressure (DBP) were measured using an aneroid sphygmomanometer and stethoscope as recommended by the American Heart Association. The patients were categorized by hypertensive and prehypertensive based on the Seventh Report of the Joint National Committee on Prevention, Detection, Evaluation, and Treatment of High Blood Pressure.

After a fasting period of at least eight hours, the intravenous blood was collected for the evaluation of biochemical markers such as FBG, total cholesterol (TC), triglyceride (TG), high-density lipoprotein (HDL), and low-density lipoprotein (LDL) by an auto-analyzer. Glycated hemoglobin (HbA1c) levels were measured in high-performance liquid chromatography.

The CVD 10-year risk was measured using sex-specific general risk prediction equations reported by D'Agostino Sr. et al. from the Framingham Heart Study. Various variables used in the scoring were age, SBP, smoking status, diabetes status, TC, and HDL [10]. The tobacco intake and smoking status were collected using a self-administered questionnaire. The patients were categorized as having high (>20%), moderate (10-20%), and low (<10%) risk of CVD based on the total risk score calculated [11]. The cut-offs for calculating the risk scores were as follows: BMI: underweight <18.4 kg/m^2^, normal 18.5-24.9 kg/m^2^, and overweight/obese >25 kg/m^2^; SBP: normal <120 mmHg, prehypertensive 120-139 mmHg, and hypertensive >140 mmHg; DBP: normal <80 mmHg, prehypertensive 80-89 mmHg, and hypertensive >90 mmHg; WC: male risk >94 cm and female risk >80 cm; WHR: male risk ≥0.90 cm and female risk ≥0.85 cm; cholesterol: normal <200 mg/dL and risk >200 mg/dL; TG: normal <150 mg/dL and risk >150 mg/dL; LDL: normal <130 mg/dL and risk >130 mg/dL; HDL: risk <40 mg/dL; and glycemic control: uncontrolled >140 mg/dL.

Statistical analysis

The data analysis was done using IBM SPSS Statistics for Windows, Version 25.0 (Released 2017; IBM Corp., Armonk, New York, United States). The data were analyzed using mean±SD. A genderwise comparison of variables was done using an unpaired Student t-test. A chi-squared analysis was conducted to find the distribution of CVD risk factors between genders to assess the relevance of the distribution of risk factors in males and females and association between 10-year cardiovascular risk assessments and cardiovascular risk variables. A p-value <0.05 was considered statistically significant.

## Results

The demographical and clinical characteristics of the study participants are shown in Table 1. The mean of the participants was 53.76±10.54 years, and among 110 cases, 52.7% were males and 47.3% were females. The mean BMI was 22.7±3.67 kg/m^2^, and SBP and DBP were 131.78±16.87 mmHg and 85.43±9.76 mmHg, respectively. The TC level was 182.54±45.29 mg/dL, and the TG level was 176.27±10.42 mg/dL. The HbA1c level was 7.6±1.34, and the FBG level was 162.54±57.42 mg/dL, respectively.

**Table 1:** Demographical and clinical characteristics of the study participants The data were shown as mean±SD BMI: body mass index; WC: waist circumference; SBP: systolic blood pressure; DBP: diastolic blood pressure; TC: total cholesterol; LDL: low-density lipoprotein; HDL: high-density lipoprotein; TG: triglyceride; FBG: fasting blood glucose

Variables	n=110 (mean±SD)
Age (years)	53.76±10.43
Gender (n, %)	
Male	58 (52.7%)
Female	52 (47.3%)
Age at onset (years)	48.7±8.28
Duration of diabetes (years)	5.54±2.64
Height (cm)	160.62±12.14
Weight (kg)	58.65±8.72
BMI (kg/m^2^)	22.7±3.67
WC (cm)	86.08±10.42
SBP (mmHg)	131.78±16.87
DBP (mmHg)	85.43±9.76
TC (mg/dL)	182.54±45.29
LDL (mg/dL)	103.65±34.28
HDL (mg/dL)	43.04±8.45
TG (mg/dL)	176.27±10.42
FBG (mg/dL)	163.54±57.42
HbA1c (%)	7.6±1.34

The genderwise comparison of demographics, anthropometrics, and clinical characteristics is shown in Table 2. The physical measurements such as height (p=0.03) and weight (p=0.02) were significantly higher in males as compared to females. Meanwhile, the BMI was higher in females as compared to males, and it was significant (23.5±3.62 vs. 22.1±3.54; p=0.03). The SBP (135.54±16.87 vs. 128.42±15.28 mmHg) was higher in males, and TC was higher in females (190.45±43.76 vs. 175.29±45.34 mg/dL; p=0.006) and found to be significant. 

**Table 2:** Genderwise comparison of demographics, anthropometrics, and clinical characteristics among diabetic patients The data were shown as mean±SD. The comparison between genders was done using an unpaired Student t-test. * denotes significant, p<0.05, while NS non-significant BMI: body mass index; WC: waist circumference; SBP: systolic blood pressure; DBP: diastolic blood pressure; TC: total cholesterol; LDL: low-density lipoprotein; HDL: high-density lipoprotein; TG: triglyceride; FBG: fasting blood glucose

Variables	Male (n=58) mean±SD	Female (n=52) mean±SD	P-value
Age (years)	53.54±10.56	53.86±10.28	0.76^NS^
Age at onset (years)	48.65±8.45	48.76±8.12	0.52^NS^
Duration of diabetes (years)	5.43±2.54	5.65±2.76	0.18^NS^
Height (cm)	167.76±13.76	153.45±12.87	0.03^*^
Weight (kg)	62.17±8.28	55.43±7.54	0.02^*^
BMI (kg/m^2^)	22.1±3.54	23.5±3.62	0.03^*^
WC (cm)	84.78±10.38	87.34±10.65	0.65^NS^
SBP (mmHg)	135.54±16.87	128.42±15.28	0.006^*^
DBP (mmHg)	86.15±9.21	84.76±9.04	0.49^NS^
TC (mg/dL)	175.29±45.34	190.45±43.76	0.001^*^
LDL (mg/dL)	103.87±33.62	104.45±33.16	0.54^NS^
HDL (mg/dL)	41.32±8.16	44.76±8.29	0.24^NS^
TG (mg/dL)	178.56±10.26	175.42±10.43	0.39^NS^
FBG (mg/dL)	164.32±56.74	162.56±56.12	0.81^NS^
HbA1c (%)	7.8±1.65	7.4±1.42	0.43^NS^

The association between gender and various categories of cardiovascular risk factors is shown in Table 3. In this study, most of the patients were in the normal weight category, representing 64.5%. Out of 110 cases, 33 were overweight, out of which the majority of them were males (19 (32.8%) and females (14 (27%)), and it was not significant (p=0.82). The increased risk of WC was higher in females when compared to males, and it was significant (86.5% vs. 13.8%; p=0.001). Based on the SBP and DBP, overall, 37.3% and 44.5% had hypertension. When considering the SBP, males are more affected by hypertension than females (55.2% vs. 17.3%; p=0.007), but for DBP, it was not significant (p=0.87). The lipid profile risk factors were as follows: elevated TC level was observed in 36.4%, LDL in 18.2%, TG in 40.9%, and decreased HDL in 40.9% of the patients, respectively. When compared between genders, elevated cholesterol was more prevalent in males as compared to females (48.3% vs. 23.1%; p=0.008), while LDL, HDL, and TG were not significant among the genders. In total, 20% of the patients were smokers or tobacco users, and the frequency was higher for males compared to females (31% vs. 7.7%; p=0.006). The glycemic control was low among the patients and was observed only in 21.8% of the cases. Meanwhile, glycemic control was more prevalent in females as compared to males (28.8% vs. 15.5%; p=0.02). The 10-year CVD risk reveals that the majority of the patients were at high risk (43.6%), moderate risk (35.5%), and low risk (20.9%). The frequency of high 10-year CD risk was higher in males as compared to females (56.9% vs. 29.8%), and females were more prone to moderate risk as compared to males (40.4% vs. 31%). 

**Table 3:** Genderwise comparison of various classes of cardiovascular risk factors among diabetic patients The data were shown as frequency (%). Chi-squared test was used for the comparison among the genders. * denotes significant, p<0.05, while NS non-significant BMI: body mass index; WC: waist circumference; SBP: systolic blood pressure; DBP: diastolic blood pressure; TC: total cholesterol; LDL: low-density lipoprotein; HDL: high-density lipoprotein; TG: triglyceride; CVD: cardiovascular disease

Risk factors	Male (n=58)	Female (n=52)	Total (n=110)	Chi-square; p-value
BMI				
Underweight	4 (6.9%)	2 (3.8%)	6 (5.5%)	X^2^=3.52; p=0.82^NS^
Normal	35 (60.3%)	36 (69.2%)	71 (64.5%)	
Overweight	19 (32.8%)	14 (27%)	33 (30%)	
WC				
Risk (male: >90 cm; female >80 cm)	8 (13.8%)	45 (86.5%)	53 (48.2%)	X^2^=30.98; p=0.001^*^
Normal	50 (86.2%)	7 (13.5%)	57 (51.8%)	
SBP				
Hypertensive	32 (55.2%)	9 (17.3%)	41 (37.3%)	X^2^=20.18; p=0.007^*^
Prehypertensive	17 (29.3%)	27 (51.9%)	44 (40%)	
Normal	9 (15.5%)	16 (30.8%)	25 (22.7%)	
DBP				
Hypertensive	28 (48.3%)	21 (40.4%)	49 (44.5%)	X^2^=2.16; p=0.87^NS^
Prehypertensive	23 (39.6%)	25 (48.1%)	48 (43.6%)	
Normal	7 (12.1%)	6 (11.5%)	13 (11.8%)	
TC				
Risk (>200 mg/dL)	28 (48.3%)	12 (23.1%)	40 (36.4%)	X^2^=22.04; p=0.008^*^
Normal (<200 mg/dL)	30 (51.7%)	40 (76.9%)	70 (63.6%)	
LDL				
Risk (>130 mg/dL)	12 (20.7%)	8 (15.4%)	20 (18.2%)	X^2^=3.45; p=0.48^NS^
Normal (<130 mg/dL)	46 (79.3%)	44 (84.6%)	90 (81.8%)	
HDL				
Risk (>40 mg/dL)	25 (43.1%)	19 (36.5%)	44 (40%)	X^2^=2.78; p=0.36^NS^
Normal	33 (56.9%)	33 (63.5%)	66 (60%)	
TG				
Risk (>150 mg/dL)	26 (44.8%)	19 (36.5%)	45 (40.9%)	X^2^=1.43; p=0.58^NS^
Normal (<150 mg/dL)	32 (55.2%)	33 (63.5%)	65 (59.1%)	
Smoking/tobacco				
Yes	18 (31%)	4 (7.7%)	22 (20%)	X^2^=25.76; p=0.006^*^
No	40 (69%)	48 (92.3%)	88 (80%)	
Glycemic control				
Yes	9 (15.5%)	15 (28.8%)	24 (21.8%)	X^2^=21.26; p=0.02^*^
No	49 (84.5%)	37 (71.2%)	86 (78.3%)	
10-year CVD risk				
Low	7 (12.1%)	16 (30.8%)	23 (20.9%)	X^2^=18.42; p=0.001^*^
Moderate	18 (31%)	21 (40.4%)	39 (35.5%)	
High	33 (56.9%)	15 (29.8%)	48 (43.6%)	

The association between different classes of cardiovascular risk factors separated by 10-year CVD risk categories is shown in Table 4. The results showed that the majority of the high-risk category was overweight (54.2%), while those in moderate risk (10.2%) and low risk (13.1%) were overweight. However, there was no significant association between BMI and the 10-year CVD risk category (p=0.75). The WC had not shown a significant association with the probability of CVD progression in the future 10 years (p=0.32). In patients with a high-risk category for 10-year CVD risk, the majority of the patients had hypertension (60.4%) when compared to moderate risk (30.8%) and low risk (8.7%), which was found to be significant (p=0.001). Meanwhile, prehypertension was higher in patients with moderate risk (48.7%) when compared to low risk (47.8%) and high risk (29.2%). The majority of the patients in the high-risk category of 10-year CVD risk were in the risk category of TC (54.2%) and TG (60.4%) as compared to patients in the moderate- and low-risk categories and were found to be significant. Smoking was the most significant risk factor in predicting the 10-year CVD risk, and the frequency was higher in high-risk patients (33.3%) as compared to moderate risk (10.3%) and low risk (8.7%), and it was significant (p=0.007). In addition, patients at high risk for the development of CVD for 10 years had uncontrolled diabetes as compared to patients at moderate and low risk (p=0.04).

**Table 4:** Association between cardiovascular risk factors and 10-year CVD risk categories The data were shown as frequency (%). Chi-squared test was used for the comparison among the genders. * denotes significant, p<0.05, while NS non-significant BMI: body mass index; WC: waist circumference; SBP: systolic blood pressure; DBP: diastolic blood pressure; TC: total cholesterol; LDL: low-density lipoprotein; HDL: high-density lipoprotein; TG: triglyceride; CVD: cardiovascular disease

Risk factors	Low risk (n=23)	Moderate risk (n=39)	High risk (n=48)	Chi-square; p-value
BMI				
Underweight	2 (8.7%)	3 (7.7%)	1 (2.1%)	X^2^=1.42; p=0.75^NS^
Normal	18 (78.2%)	32 (82.1%)	20 (41.7%)	
Overweight	3 (13.1%)	4 (10.2%)	26 (54.2%)	
WC				
Risk (male: >90 cm; female: >80 cm)	13 (56.5%)	18 (46.1%)	22 (45.8%)	X^2^=0.96; p=0.32^NS^
Normal	10 (43.5%)	21 (53.8%)	26 (54.2%)	
SBP				
Hypertensive	2 (8.7%)	12 (30.8%)	29 (60.4%)	X^2^=25.74; p=0.001^*^
Prehypertensive	11 (47.8%)	19 (48.7%)	14 (29.2%)	
Normal	10 (43.5%)	8 (20.5%)	5 (10.4%)	
DBP				
Hypertensive	10 (43.5%)	17 (43.6%)	22 (45.8%)	X^2^=1.28; p=0.18^NS^
Prehypertensive	7 (30.4%)	18 (46.2%)	24 (50%)	
Normal	6 (26.1%)	4 (10.2%)	3 (6.2%)	
TC				
Risk (>200 mg/dL)	3 (13%)	11 (28.2%)	26 (54.2%)	X^2^=15.65; p=0.03^*^
Normal (<200 mg/dL)	20 (87%)	28 (71.8%)	22 (45.8%)	
LDL				
Risk (>130 mg/dL)	3 (13%)	7 (17.9%)	10 (20.8%)	X^2^=2.17; p=0.65^NS^
Normal (<130 mg/dL)	20 (87%)	32 (82.1%)	38 (789.2%)	
HDL				
Risk (>40 mg/dL)	8 (34.8%)	16 (41%)	26 (54.2%)	X^2^=2.98; p=0.54^NS^
Normal	15 (65.2%)	23 (59%)	22 (45.8%)	
TG				
Risk (>150 mg/dL)	4 (17.4%)	12 (30.8%)	29 (60.4%)	X^2^=8.92; p=0.03^*^
Normal (<150 mg/dL)	19 (82.6%)	27 (69.2%)	I9 (39.6%)	
Smoking/tobacco				
Yes	2 (8.7%)	4 (10.3%)	16 (33.3%)	X^2^=16.23; p=0.007^*^
No	21 (91.3%)	35 (89.7%)	32 (66.7%)	
Glycemic control				
Yes	14 (60.9%)	9 (23.1%)	3 (6.3%)	X^2^=7.45; p=0.04^*^
No	9 (39.1%)	30 (76.9%)	45 (93.7%)	

## Discussion

The current study has examined the 10-year cardiovascular risk in T2DM patients and the factors involved in the risk progression using the Framingham CVD risk scores. Given the significant health implications associated with CVD, the Framingham CVD risk scores have emerged as a prevalent approach for forecasting the incidence of CVD within a decade. The efficacy of the Framingham CVD risk scores in this context has been published [12]. Research has also demonstrated that as the risk of getting CVD increases, the effectiveness of the intervention required also increases. Multiple trials focused on primary prevention have provided evidence of a notable impact of interventions on cardiovascular events among those with lower risk scores [13]. In the present study, based on the Framingham CVD risk scores, the majority of the diabetic patients are in the high-risk category, encompassing 43.6%, followed by moderate in 35.5% and low-risk in 20.9% of the patients. Likewise, in a recent study done by Unnikrishnan et al., 60.5% of newly diagnosed T2DM patients had a high risk for CVD in the next 10 years [14]. In contrast, Maharana et al. reported a low incidence of the high-risk category in 11.1%, moderate risk in 33.05%, and low risk in 55.93% of the patients [15]. The large proportion of high-risk patients in our study might be due to various factors such as male gender, increased cholesterol and TG levels, uncontrolled diabetes, smoking, and tobacco use.

In the present study, we have analyzed the genderwise difference in the risk factors among diabetic patients. A high proportion of females showed increased WC (86.5% vs. 13.8%; p=0.001) when compared to males. Meanwhile, males showed a high proportion of hypertension (55.2% vs. 17.3%; p=0.007), increased TC (48.3% vs. 23.1%; p=0.008), and smoking and tobacco use (31% vs. 7.7%; p=0.006). Similarly, in the Unnikrishnan et al. study, the incidence of hypertension (44.9 vs. 37.4%, p<0.001), smoking (28.6% vs. 8.4%, p<0.001), and cholesterol abnormality (21.8% vs. 10.0%, p<0.001) is higher in males as compared to females [14]. In another study done by Davalagi et al. hypertension (63.8% vs. 33.7; p<0.001) and smoking habit (62.6 vs. 19.1; p<0.001) were more prevalent in men as compared to women. One potential reason for the disparities in risk factors between diabetic men and women could be hormonal differences [16]. Studies have shown that estrogen may have a protective effect on the cardiovascular system, while testosterone may have a detrimental effect. This could explain why women with diabetes tend to have different risk factors for heart disease compared to men [17]. Additionally, differences in lifestyle factors such as diet, exercise, and stress management may also play a role in the differences observed between genders. 

In the present study, the significant risk factor for the high-risk category in the Framingham risk score is hypertension, in which 60.4% of patients in high risk had hypertension as compared to the low- and moderate-risk category. In the context of unadjusted analysis and adjustment models of major cardiovascular risk factors, it was observed that individuals with both T2DM and hypertension exhibited a heightened susceptibility to both cardiovascular events and stroke as that of the patients with T2DM alone [18]. These findings indicate that hypertension is associated with an increased susceptibility to CVD.

The present study showed that elevated cholesterol levels were associated with a high Framingham risk score. About 54.2% of the patients with elevated cholesterol levels are in the high-risk category when compared to the moderate- and low-risk categories. In a study done by Khil et al., elevated TC levels have a significant association with the development of CVD in diabetic patients [19]. 

The current study also revealed that in the Framingham risk score high-risk category, there is a higher proportion of patients, 33.3%, who smoke or use tobacco as compared to patients with moderate and low risk. Likewise, Ikhsan et al. showed a high prevalence of current smoking in patients in the high-risk category, encompassing 65.7%, and it was significant (p=0.009) [20]. Smoking elevates the likelihood of developing CVD through direct damage to the endothelium caused by toxic substances present in cigarettes, such as carbon monoxide and nicotine. These substances lead to the formation of blebs on the surface of the artery, the release of endothelial cells (endothelial damage), alterations in platelets, elevated levels of fibrinogen and C-reactive protein, and the induction of proinflammatory cytokines [21]. In addition, T2DM increases blood viscosity, resulting in impaired blood flow to tissues and an increased susceptibility to CVD [22].

We have also observed that in the Framingham risk score high-risk category, the majority of cases have uncontrolled diabetes, constituting 93.7%, as compared to the moderate- and low-risk categories. In a recent study done by Fayed et al., a large proportion of patients with uncontrolled diabetes are in the high-risk category of the Framingham risk score when compared to patients with controlled diabetes, and it was significant (32.2% vs. 16.8; p<0.01) [13]. A previous study revealed that uncontrolled diabetic patients had an increased risk of developing CVD [23]. Further, for a one-unit increase in HbA1c, there is an 18% increase in the development of MI and stroke.

Limitations

The study has certain limitations; this was a single-center study with a very short duration study, which affects the generalizability of the results and has a smaller sample size. The study has not evaluated diabetic patients with existing or prior CVD. Furthermore, the study did not evaluate newly diagnosed diabetic subjects.

## Conclusions

The present study concluded that the majority of T2DM patients are at high risk of developing CVD. In addition, a significant proportion of individuals living with diabetes exhibited elevated cardiovascular risk factors, with a greater prevalence observed among males in comparison to females. Smoking and tobacco use, hypertension, elevated TC, and uncontrolled diabetes are the important risk factors involved in the progression of cardiovascular disorders. The Framingham risk score might be a useful model to predict the future CVD risk among diabetic patients. 
